# Oxidative stress alleviating potential of galactan exopolysaccharide from *Weissella confusa* KR780676 in yeast model system

**DOI:** 10.1038/s41598-022-05190-2

**Published:** 2022-01-20

**Authors:** Digambar Kavitake, Bhavana Veerabhadrappa, S. J. Sudharshan, Sujatha Kandasamy, Palanisamy Bruntha Devi, Madhu Dyavaiah, Prathapkumar Halady Shetty

**Affiliations:** 1 Department of Food Science and Technology, Pondicherry University, Pondicherry, 605014 India; 2 Department of Biochemistry and Molecular Biology, Pondicherry University, Pondicherry, 605014 India

**Keywords:** Biochemistry, Biotechnology, Microbiology

## Abstract

In the present study, galactan exopolysaccharide (EPS) from *Weissella confusa* KR780676 was evaluated for its potential to alleviate oxidative stress using in vitro assays and in vivo studies in *Saccharomyces cerevisiae* (wild type) and its antioxidant (*sod1∆, sod2∆, tsa1∆, cta2∆* and *ctt1∆*), anti-apoptotic (*pep4∆* and *fis1∆*) and anti-aging (*sod2∆*, *tsa1∆* and *ctt1∆*)) isogenic gene deletion mutants. Galactan exhibited strong DPPH and nitric oxide scavenging activity with an IC_50_ value of 450 and 138 µg/mL respectively. In the yeast mutant model, oxidative stress generated by H_2_O_2_ was extensively scavenged by galactan in the medium as confirmed using spot assays followed by fluorescencent DCF-DA staining and microscopic studies. Galactan treatment resulted in reduction in the ROS generated in the yeast mutant cells as demonstrated by decreased fluorescence intensity. Furthermore, galactan exhibited protection against oxidative damage through H_2_O_2_ -induced apoptosis inhibition in the yeast mutant strains (*pep4∆* and *fis1∆*) leading to increased survival rate by neutralizing the oxidative stress. In the chronological life span assay, WT cells treated with galactan EPS showed 8% increase in viability whereas *sod2∆* mutant showed 10–15% increase indicating pronounced anti-aging effects. Galactan from *W. confusa* KR780676 has immense potential to be used as a natural antioxidant for nutraceutical, pharmaceutical and food technological applications. As per our knowledge, this is the first report on in-depth assessment of in vivo antioxidant properties of a bacterial EPS in a yeast deletion model system.

## Introduction

Oxidation is an essential process to maintain the biological processes and also for the production of energy in all living organisms^1^. Reactive oxygen species (ROS) and reactive nitrogen species (RNS) radicals are produced from normal catabolism of oxygen and nitrogen molecules respectively. Severe oxidative stress leads to various degenerative conditions like DNA damage, cellular degeneration and carcinogenesis. These may result in many health impairments such as aging, cardiovascular diseases, cancer, cirrhosis, atherosclerosis, diabetes and rheumatoid arthritis^2–6^. Antioxidants are the molecules which scavenge free radicals generated in food or living system and leading to the prevention of oxidative damage related health conditions^7–9^. Although many synthetic antioxidants are available as strong radical scavengers, some of them have been linked to some undesirable side effects^10^. In view of this, there has been increased interest and demand for natural antioxidants in the use of food and pharma industries.

Most of the plant and mushroom-based polysaccharides have been reported as significant protective agents against ROS^11–21^. Various microbial exopolysaccharides (EPS) including from lactic acid bacteria (LAB) have also been reported for their substantial antioxidant properties. These are considered to be safer alternatives to the synthetic ones. In the recent years, many EPS from LAB have been studied for their antioxidant potential and prevention of oxidative damage^22,23^. Antioxidant potential and protective role of Weissella EPS have also been reported from a variety of EPS isolated from Weissella many strains like *Weissella confusa* EPSWWC, *W. confusa* OF126, *W. cibaria* GA44, *W. cibaria* YB-1, *W. confusa* W4 and *W. cibaria* SJ14^24–29^. In vivo antioxidant properties can be studied using various model systems such as cell lines^30^, *C. elegans*^31^, yeast^32^ and mice^33^. Previously, various compounds have been screened for their potential antioxidant properties using *Saccharomyces cerevisiae* yeast gene deletion mutant model system^32,34–37^.

This study is focused on the antioxidant potential of galactan EPS produced by the probiotic strain *Weissella confusa* KR780676 from an Indian traditional fermented food (*Idli batter*)^38,39^. In this paper, the galactan EPS is screened for in vitro antioxidant potential using DPPH, nitric oxide and hydroxyl radical scavanging assays and in vivo antioxidant, anti-apoptotic and anti-aging properties using yeast gene deletion mutant model system.

## Materials and methods

All the chemicals including media supplements were procured from Hi-Media Laboratories Pvt. Ltd., India, and DCF-DA (2,7 Dichlorodihydrofluorescein diacetate) from Sigma.

### Microbial culture

*Weissella confusa* KR780676 isolated from an Indian acidic fermented food (*Idli batter*), reported to produce galactan EPS was used in this present study^38^.

### Detection of EPS production

The EPS production of *W. confusa* was observed from colony level. Briefly, the strain was cultured on MRS agar (supplemented with 2% sucrose). After 48 h at 30 °C, appearance of slimy/mucous colonies was observed. Production of galactan EPS was further verified under scanning electron microscopy (SEM) analysis.

### Extraction of EPS

EPS extraction process was performed as per the method described in Kavitake et al.^38^. Fresh inoculum (10%) of *W. confusa* was added to 2% sucrose enhanced MRS medium at 30 °C for 48 h under static condition. The suspension was in this manner centrifuged (12,000×*g* for 15 min) to isolate the biomass and further treated with tri-chloro acidic acid to eliminate the protein moieties. The galactan-EPS was precipitated using ice cold ethanol (threefold the volume), centrifuged (19,200×*g* for 15 min) and the resultant EPS was dissolved in Milli-Q water. The crude EPS was dialyzed at 12–14 kDa (48 h, 4 °C) and freeze dried by lyophilization for 48 h.

### In vitro antioxidant properties of galactan EPS

DPPH assay for galactan was executed according to the earlier report by Ye et al.^9^ and percent scavenging activity (%) was calculated by the following equation.

$$DPPH\;scavanging\;activity\;\left( \% \right) = \frac{{Ao - As}}{{Ao}}\; \times \;100$$

where *Ao* and *As* is the absorbance of the control (blank, without EPS) and sample respectively.

Nitric oxide (NO) radical scavenging assay for galactan was performed according to Sreejayan et al.^40^ and calculated as following equation,

$$NO\;scavanging\;activity\;\left( \% \right) = \frac{{Ao - As}}{{Ao}}\; \times \;100$$

where *Ao* is the absorbance of the control (blank, without EPS) and *As* is the absorbance in the presence of the EPS.

Reducing power activity was measured for galactan EPS as reported by Ye et al.^9^. The absorbance was read at 700 nm and the reductive potential is indicated by a high absorbance capacity of the reaction mixture. Ascorbic acid (Vc) was used as a positive control.

Hydroxyl radical scavenging activity of galactan EPS was evaluated as described by Yang et al.^41^. The absorbance was read at 536 nm and scavenging percentage was calculated as:

$${\text{The scavenging activity (\% )}} = \left[ {\frac{{1 - \left( {A_{{Sample}} - A_{{Blank}} } \right)}}{{A_{{Control}} }}} \right] \times 100$$

where *A *_*sample*_ is the absorbance of the sample, *A *_*blank*_ is absorbance in the absence of a sample and H_2_O_2_ solution, and *A *_*control*_ is an absorbance in the absence of the sample.

### In vivo antioxidant properties of galactan EPS in yeast mutant strains

The Yeast, *S. cerevisiae*, BY4741 wild type (WT) (MATa *his3*∆:*leu2∆:met15∆:ura3∆*) and gene deletion mutant strains were procured from Thermo Fisher Scientific, USA. Yeast strains were grown in yeast peptone dextrose (YPD) medium supplemented with or without 200 µg/mL of Geneticin (G418 sulfate) for the selection of mutants. YPD solid medium was prepared by the addition of 2% Bacto agar to the YPD liquid medium^42^.

#### Effect of EPS on the yeast growth

Exponentially growing yeast wild type (WT) culture (approximately 1 × 10^4^ cells) was treated with different concentrations (0–400 μg/mL) of EPS in a microwell plate and the final volume was made up to 200 μL with YPD broth. The culture was incubated for 18 h at 30 °C followed by serial dilution and spreading on YPD agar plates. The plates were incubated at 30 °C for 2 days and colony forming units (CFUs) were counted and the viability was expressed as percent CFU^43^.

#### Measurement of biomarkers of oxidative stress

Exponentially growing yeast WT cells were pre-treated with or without 300 μg/ml of EPS for 2 h. Then the cells were exposed to 1 mM H_2_O_2_ for 1 h at 30 °C in a shaker incubator and processed for the measurement of SOD activity and lipid peroxidation levels as per the method described in earlier reports^44–46^.

### Antioxidant property of EPS in *S. cerevisiae* gene deletion mutants

Exponentially growing cultures of yeast WT and antioxidant-deficient mutant strains (*sod1∆*, *sod2∆*, *tsa1∆*, *cta1∆*, *ctt1∆*, *glr1∆* and *yhb1∆*) were treated with 300 μg/mL EPS for 2 h followed by exposed to 1 mM H_2_O_2_ for 1 h. Serially diluted cells were spread on YPD agar plates, incubated for 2 days at 30 °C and viability was calculated. For spot assay, cultures were serially diluted in 10-folds, 4 μL of which was spotted on YPD agar plates and incubated at 30 °C for 2 days and photographed^43,47^.

#### Detection and measurement of ROS

Exponentially growing WT and antioxidant-deficient mutant strains (*sod1∆*, *sod2∆*, *tsa1∆*, *cta1∆* and *ctt1∆*) were pretreated with or without EPS for 2 h and exposed to 1 mM H_2_O_2_ for 1 h at 30 °C. Cell pellets after centrifugation at 5000 rpm for 5 min were washed twice with PBS buffer, re-suspended in 200 μL of PBS and incubated with 20 μM DCF-DA in dark for 15–20 min at room temperature. Immediately after the incubation, cells were washed twice with PBS, mounted on the slides and observed under Olympus Ix71 fluorescence microscope under 40 × objective using blue filter. For quantification of ROS, DCF-DA stained cells were resuspended in 200 μL of PBS after washing and the intensity of DCF fluorescence was measured using a spectrofluorometer at the excitation maximum and emission wavelengths of 495/529 nm. Fluorescence units were plotted against each treated and untreated culture and compared^48,49^.

### Anti-apoptotic activity of galactan

#### Spot and CFU assays

Exponentially grown WT and anti-apoptotic-deficient mutant (*pep4∆* and *fis1∆*) cells were pre-treated with EPS and incubated along with respective untreated controls for 2 h. For CFU counts, cultures were treated or untreated with EPS for 2 h and incubated with 0.5 mM H_2_O_2_ for 1 h. Each serially diluted culture was spread on YPD agar plates, incubated at 30 °C for 2 days and cell viability was represented as percent CFU. For spot assay, cultures were allowed for serial dilution followed by spotting on YPD agar plates with or without 1 mM H_2_O_2_. Plates were incubated at 30 °C for 2 days and photographed^47^.

#### Detection of an anti-apoptotic marker of EPS using yeast mutant strains

To further confirm the rescue action of EPS on yeast cells from the apoptotic cell death induced by hydrogen peroxide in yeast, WT and anti-apoptotic-deficient mutant strains (*pep4∆* and *fis1∆*) were examined for apoptosis markers. Exponentially growing WT, *pep4∆* and *fis1∆* cells were treated or untreated with 300 µg/mL EPS for 2 h and then were exposed to 1 mM H_2_O_2_ for 1 h. Both treated and untreated cells were stained with acridine orange and ethidium bromide (AO/EB) and observed under a fluorescent microscope for chromatin condensation^50,51^.

For DAPI staining**,** the treated and untreated cells were fixed with 4% paraformaldehyde and incubated with 1 μg/mL DAPI for 5–10 min in dark at room temperature. Cells were mounted on the slides after washing with PBS and observed under Olympus IX71 fluorescence microscope (UV filter, 40 × objective) for nuclear fragmentation^52,53^.

### Anti-aging effect of EPS by chronological lifespan assay

Yeast wild type and antioxidant deficient mutant (*sod2∆*, *tsa1∆* and *ctt1∆*) cultures were grown to reach the stationary phase and incubated with or without EPS for chronological lifespan (CLS) assay. The survivability of every strain was calculated at different time intervals from 0 to 30 days. Cell viability was expressed as percent CFU for both treated and untreated cultures^54,55^.

### Statistical analysis

All the experiments were carried out in triplicates (± SD) and statistically analyzed using IBM SPSS 20 software in a one-way ANOVA model. Tukey’s HSD comparison test (*p* < 0.05) was used to measure the significance level.

## Results and discussion

Lactic acid bacteria (LAB) isolated from fermented foods have fetched enormous attention of the food technologists over the last few years because of their proven probiotic properties. Among the bacteria isolated from Indian fermented foods are *Lactobacillus spp.*, *Lactococcus spp.*, *Leuconostoc spp.* and the less explored *Weisella spp*. Like the other beneficial LABs, the *Weissella confusa* strain KR780676 which was isolated from fermented idli batter in our lab has been reported to act as a potential probiotic candidate^39^.

Earlier reports, this galactan has been characterized as a linear homopolysaccharide and also screened for its physico-chemical, functional and emulsifying properties^38,56–58^. We have also reported that the cells and cell supernatants of *W. confusa* KR780676 showed strong anti- oxidant activity^39^.

**Figure 1:**
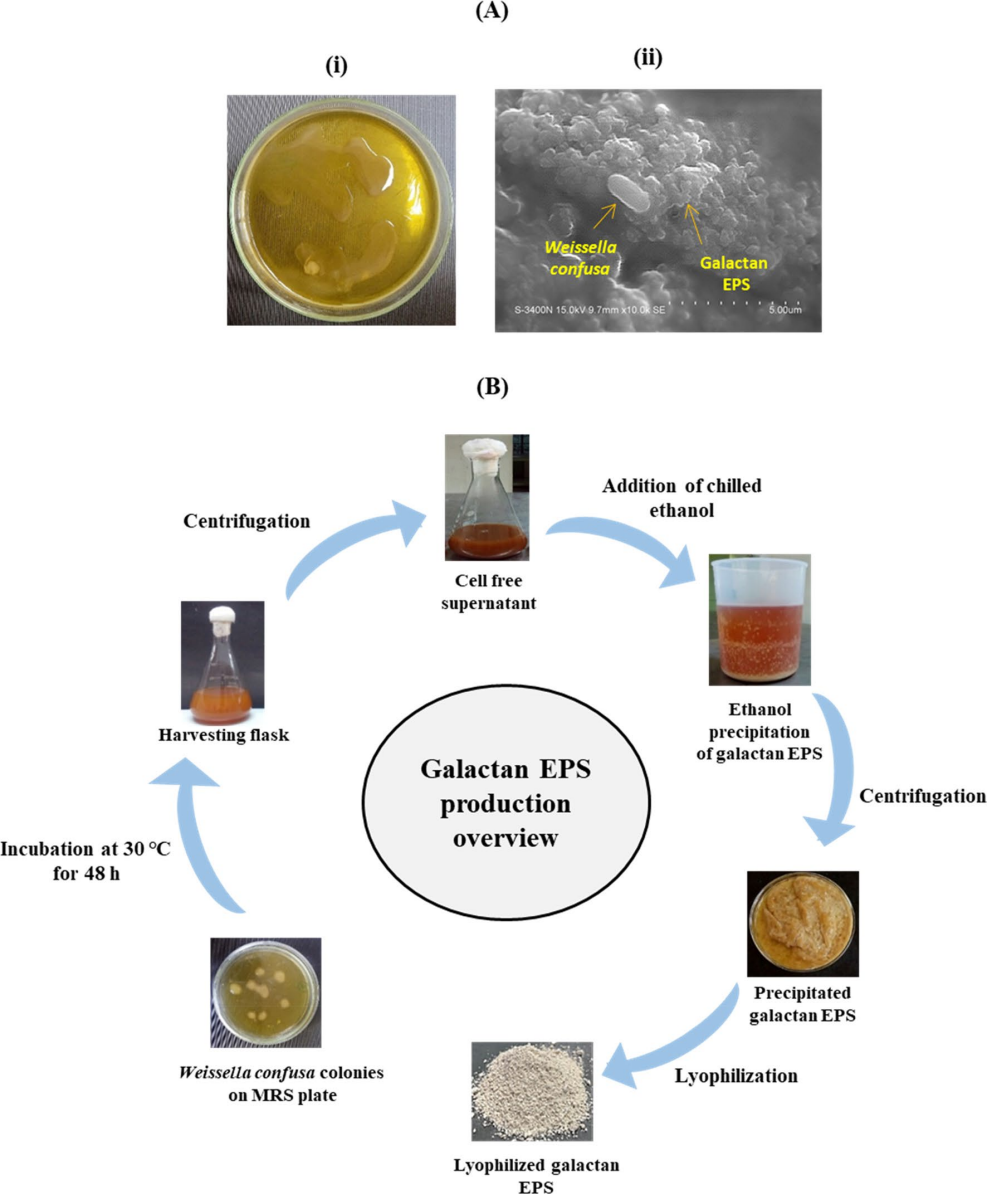
(**A**) Detection of galactan EPS production from *Weissella confusa* KR780676: (**i**) Weissella slimy colonies on MRS agar enriched with 2% sucrose, incubated for 48 h (**ii**) SEM analysis of Weissella colony showing presence of galactan EPS along with cells. (**B**) Overview of galactan EPS production.

EPS production from *W. confusa* KR780676 was observed on MRS agar plate enriched with 2% sucrose, incubated for 48 h (Fig. 1A-i), which revealed Weissella slimy colonies and it was further confirmed in SEM image (of the Weissella colony) showing presence of galactan EPS along with cells (Fig. 1A-ii). Step by step EPS production is overviewed with the pictoral representation in the Fig. 1B.

### In vitro antioxidant properties of galactan EPS

**Figure 2:**
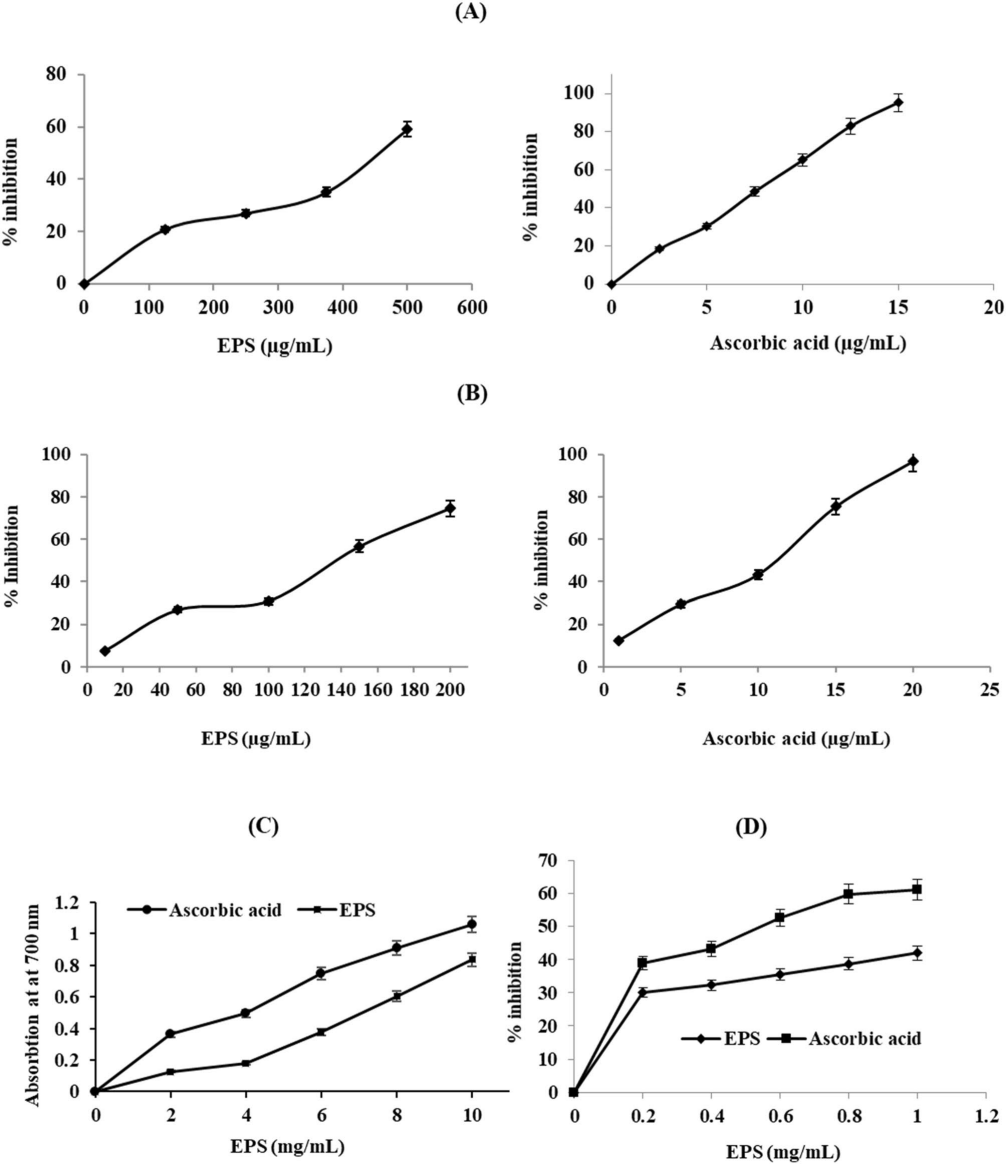
In vitro antioxidant properties of galactan. (**A**) DPPH radical scavenging activity, (**B**) Nitric oxide assay, (**C**) Reducing power assay and (**D**) Hydroxyl radical scavenging activity of galactan EPS.

As shown in Fig. 2A, the DPPH scavenging activity is observed to be concentration-dependent, the scavenging activity increased with the galactan concentration. The half-maximal effective concentration (IC_50_) of ascorbic acid was 8.8 µg/mL, while galactan EPS was 450 µg/mL. 1,1-diphenyl-2-picrylhydrazyl (DPPH) is a stable free radicle which delocalizes the unpaired electrons against the molecule as a whole, thus prevents the molecules from dimerization and results in deep violet color^59^. When DPPH solution is added to different concentrations (50 to 500 µg/mL) of galactan, it gives rise to a reduction of violet color as the concentration increased. Antioxidants when reacting with an electron of DPPH, the deep violet color of DPPH turns to light violet color; and the intensity of the color depends on the antioxidant activity of the substrate^60^. Galactan showed 60% DPPH scavenging potential which is higher than EPS from endophytic bacterium *Paenibacillus polymyxa* EJS-3 (< 45.40%)^33^.

Nitric oxide is produced when sodium nitroprusside is decomposed in an aqueous solution at a pH of 7.2. Nitrate and nitrite are produced under aerobic conditions when nitric oxide binds with oxygen^61^. As shown in Fig. 2B, galactan has a potential to reduce nitric oxide generation from sodium nitroprusside as the half-maximal effective concentration (IC_50_) is 138 µg/mL, while standard ascorbic acid was 11 µg/mL.

As shown in Fig. 2C, the reducing power activity of EPS is positively correlated with its concentration in increasing order. Standard (ascorbic acid) showed higher reducing capacity than galactan EPS which was in agreement with Ye et al.^9^. The reducing power activity of galactan indicates the potential antioxidant activity. When potassium ferricyanide [K_3_Fe (CN)_6_] is added in galactan EPS, the antioxidants present in it reduces into potassium ferricyanide [K_4_Fe(CN)_6_].

Hydroxyl radical scavenging activity of galactan is shown in Fig. 2D. Galactan showed 41.93% as the highest activity, whereas 58.10% by the standard at the same concentration (1 mg/mL). Results trend is in agreement with previous reports for EPS from *Lactobacillus plantarum* C88 (85.21% for 4 mg/mL concentration of EPS)^62^ and *Paenibacillus polymyxa* EJS-3 (68.55% for 1 mg/mL concentration of EPS)^63^. Compare to standards, galactan showed 72.16% efficiency whereas EPS from *Lactobacillus plantarum* C88^62^ and *Paenibacillus polymyxa* EJS-3^63^ showed 95.19 and 68.55% efficiency respectively.

### In vivo antioxidant properties of galactan EPS in yeast model

Earlier reports have shown different health promoting biological properties such as anti-proliferative, anti-ulcer, cholesterol-lowering, antioxidant, anti-inflammatory and immunomodulatory activities of EPS derived from LAB^64^. In this view, it becomes important to evaluate the indetail antioxidant effect by the EPS that could support to their prebiotic and/or probiotic potential, mainly to maintain the gut homeostasis by alleviating the oxidative stress. The cellular antioxidant machinery plays a critical role in alleviating the unavoidable ROS generated through crucial cellular metabolic pathways. The imbalance between the cell’s innate antioxidant defense and the ROS generated can be very detrimental to the cells. Severe oxidative stress causes potential damage to cellular vital components, proteins, nucleic acids and lipid molecules which in turn affects many essential signalling pathways, and induces apoptosis. The dietary intake of antioxidants has been shown to lower the frequency of cellular damage markers such as ROS levels, ROS mediated DNA damage, apoptosis and cellular transformation which further results in the lowered incidence of the age associated disorders^65^ Microbial EPS like xanthan and levans have demonstrated anti-oxidant activity in in vitro assays (DPPH and hydroxyl radical assays)^5^ and against human gastric cancer cells BGC-823, respectively^66^. Previously, In this study, we have evaluated the antioxidant, antiapoptotic and antiaging effects exerted by galactan EPS isolated from *W. confusa* KR780676 using yeast *Saccharomyces cerevisiae* BY4741 as a model organism.

#### Effect of EPS on the yeast growth

**Figure 3:**
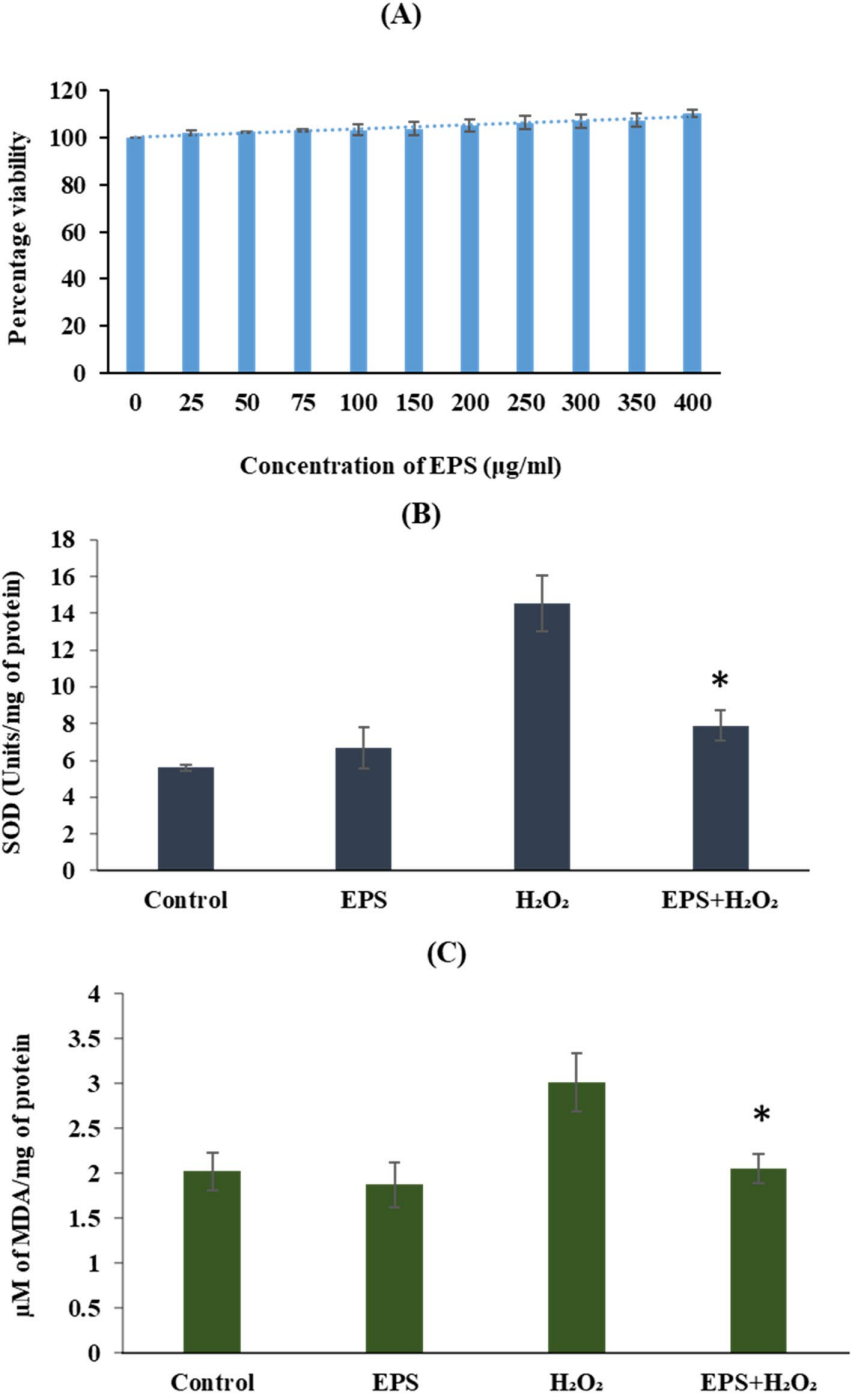
Effect of EPS on yeast and measurement of oxidative stress markers. (**A**) Effect of galactan on the yeast growth: Exponentially grown wild type (WT) cells were treated with different concentrations of galactan overnight. Cells were serially diluted, CFU assay was performed. (**B**) SOD activity: Yeast cells treated with or without EPS for 2 h followed by treatment with or without H_2_O_2_ for 1 h and performed SOD activity. (**C**) Lipid peroxidation: Yeast cells treated with or without EPS for 2 h followed by treatment with or without H_2_O_2_ for 1 h and carried out lipid peroxidation by TBARS method. Data are mean ± SD of three independent experiments. ∗represents *P* < 0.0001 a significant increase/decrease in EPS + H_2_O_2_ treated samples compared to those treated with H_2_O_2_ alone.

Wild type yeast cells treated with different concentrations of EPS did not show any growth defects confirming that the galactan did not induce any cytotoxicity or growth defects at any of concentrations ranging from 0 to 400 μg/mL (Fig. 3A). Cells treated with 300 μg/mL or higher concentration of galactan showed significantly higher growth indicating that galactan had yeast growth stimulatory activity. Galactan concentration of 300 μg/mL is used for subsequent experiments with yeast strains.

#### Galactan reduces SOD enzyme activity

Following the strong in vitro antioxidant activity displayed by galactan EPS, further subjected to check its effect on SOD activity in the yeast WT cells. Our results (Fig. 3B) show that the oxidative stress induced by H_2_O_2_ treatment caused a sharp increase in the SOD activity of H_2_O_2_ treated WT cells compared to untreated cells. In contrast, galactan pre-treatment of WT cells followed by exposure to H_2_O_2_ resulted in a ~ twofold reduction in the SOD activity compared to that of the cells treated by H_2_O_2_ alone indicating galactan helps the yeast cells in managing the superoxide radical induced oxidative stress^47,67^.

Oxidative stress induced by peroxide treatment activates the superoxide enzyme and change in the level of SOD activity is a direct measure of the level of cellular oxidative stress. In this study, NBT reduction method was used, where, NBT is an indicator of superoxide radical production. Inhibition of NBT reduction is a direct measure of SOD. Since SOD competes with NBT for superoxide radical generated by exposing riboflavin to visible light in the presence of oxygen and methionine, which is an electron donor. Superoxide reduces NBT to a blue color product, formazan which can be colorimetrically measured at 560 nm.

Previous reports indicate that LAB EPS have exerted antioxidant effect in a concentration dependent manner in in vitro assays, and in colon cancer cell lines and in vivo models. The ability of EPS to scavenge the ROS might be attributed to their diverse chemical groups^68^. Other of EPS (500 µg/ml) from *Bacillus amyloliquefaciens* have been shown to greatly influence SOD activity and protect the HepG2 cells from oxidative stress induced by H_2_O_2_^68^. Similarly, the EPS from *L. plantarum* showed a concentration dependent effect on SOD activity in Caco2 cells against H_2_O_2_ induced oxidative stress^69^. Our results, in concordant with these reports show that galactan EPS pre-treatment scavenges the superoxide radicals induced by H_2_O_2_ treatment in yeast WT cells.

#### EPS decreases cellular lipid peroxidation

Malondialdehyde (MDA) is the most studied cellular lipid peroxidation biomarker that indicates the level of oxidative stress. MDA is a chemically stable, highly reactive dialdehyde and can readily bind to proteins, nucleic acids and lipoproteins. It is highly mutagenic and can greatly affect the biochemical properties of these biomolecules which is deleterious to various signaling pathways^70^. An increase in MDA levels is an indicator of ROS-induced tissue damage and its increased levels are detected in several human pathologies^46^. In this study, to examine how EPS pre-treatment influences the lipid peroxidation induced by H_2_O_2_ in yeast WT cells, MDA levels were estimated. Results showed an approximately 1.5- fold reduction in the MDA level in EPS pre-treated cells, compared to those exposed to H_2_O_2_ alone as shown in Fig. 3C.

Previously, EPS from *L. plantarum* C88 have been shown to reduce MDA levels induced by H_2_O_2_ treatment, in a dose dependent manner (50–200 ug/ml) in Caco2 cells, indicating that the peroxide induced membrane injury of the intestinal cells can be alleviated by the supplementation of EPS^69^ (zhang 2013). Further, 500 ug/mL of EPS from *B. amyloliquefaciens* significantly reduced H_2_O_2_ induced MDA in HepG2 cells^71^. Our results on H_2_O_2_ induced MDA levels in yeast cells and its significant reduction following pre-tretment of the cells with galactan EPS indicate that the treatment with galactan EPS reduces cellular ROS induced lipid peroxidation and in turn, may protect the cells from tissue damage caused by peroxide radicals and aid in maintaining cellular integrity.

### Galactan protects yeast antioxidant gene mutants under oxidative stress

**Figure 4:**
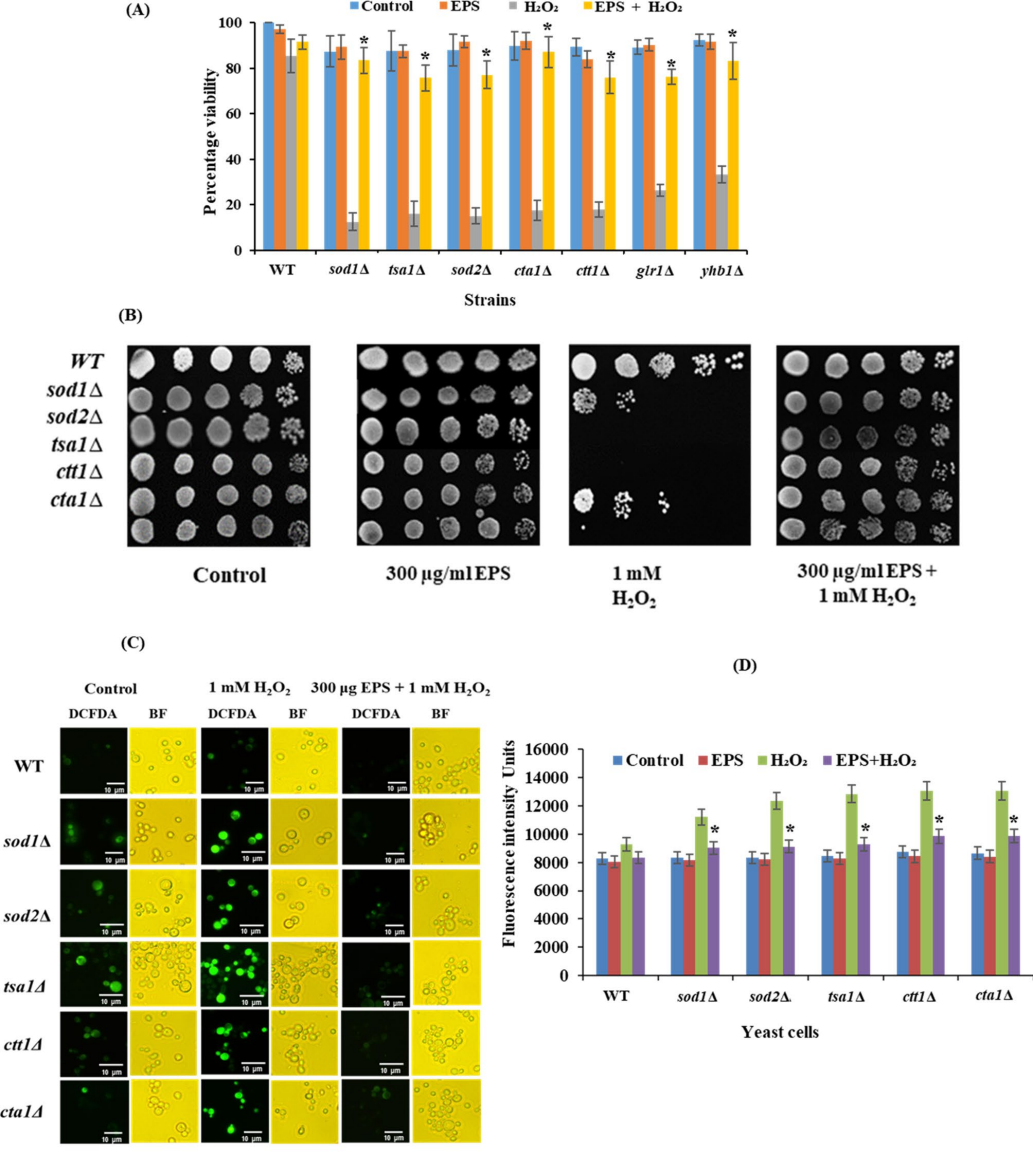
Growth protection to *S. cerevisiae* gene deletion mutants by galactan EPS from H_2_O_2_ induced oxidative stress. (**A**) Viability assay: Cells pre-treated with or without EPS for 2 h and incubated with or without H_2_O_2_ for 1 h. Then performed CFU assay. (**B**) Spot assay: Cells were serially diluted and spotted on the YPD plate. (**C** and **D**) Measurement of ROS measurement by H_2_DCF-DA.: Cells were treated with H_2_DCF-DA for 10–15 min in dark, washed and fluorescence intensity was measured using a spectrofluorometer and later images were taken using a fluorescence microscope. Data are mean ± SD of three independent experiments. ∗represents *P* < 0.0001 a significant decrease in EPS + H_2_O_2_ treated samples compared to those treated with H_2_O_2_ alone. Images shown are of at least three independent experiments.

In order to evaluate the antioxidant ability of galactan, different antioxidant gene deficient yeast mutants (that lack various oxidative stress response genes) were treated with galactan and then exposed to a sub-lethal dose of H_2_O_2_^42^. SOD (*sod1∆* and *sod2∆*), catalase (*cta1∆* and *ctt1∆*), thioredoxin peroxidase (*tsa1∆*), glutathione reductase (*glr1∆*) and nitric oxide oxidoreductase (*yhb1∆*) mutants when treated with H_2_O_2_. showed low survival (*sod1∆* 10.8%, *sod2∆* 13.17%*, tsa1∆* 13.55%*, cta1∆* 15.34%, *ctt1∆* 17.06%, *glr1∆* 27.37% and *yhb1∆* 33.87%) against WT. In contrast, with the galactan pre-treatment, the tolerance against oxidative stress increased in all the antioxidant gene-deficient mutants and their viability increased significantly (*sod1∆* 78.43%, *sod2∆* 59.96%, *cta1∆* 87%*, ctt1∆* 87%*, tsa1∆* 72.73%*, glr1∆* 75.26% and *yhb1∆* 82.41%) as shown in Fig. 4A. These results suggest that galactan can effectively scavenge the free radicals induced by H_2_O_2_ in mutants lackingspecific oxidative stress response genes and protects the cells against oxidative stress. Similar protection was observed in the spot assay, where galactan rescued the mutants from oxidative stress and increased the viability as shown in Fig. 4B.

Cells are evolved with enzymatic antioxidant defense system to withstand the endogenous oxidative stress caused by ROS generated through various physiological reactions, which would otherwise have deleterious effects on the well being of the cell. Previous reports and our results in this study suggest that EPS are able to scavenge the ROS and improve cell viability. Our results with yeast oxidative stress response mutant strains suggest that galactan EPS treatment scavenges both cytosolic and mitochondrial superoxide radicals induced by H_2_O_2_ treatment, as evidenced by the increased viability of yeast *sod1∆* and *sod2∆* mutant strains respectively. SODs are primary enzymatic antioxidant defenses in the cells against endogenous and exogenous oxidative stress and mutations in these genes have been implicated in cancer and degenerative disorders. Likewise, the antioxidant rescue of *cta1∆* and *ctt1∆* (catalase mutants) by EPS treatment indicates that treatment with galactan EPS provides protection against peroxisomal and cytosolic oxidative stress induced by H_2_O_2_. Catalase is responsible for cellular detoxification of hydrogen peroxide and its deficiency is associated with the onset of age related disorders. *TSA1*, thioredoxin peroxidase is both ribosomal associated and free cytoplasmic protein that relieves the cells from hydrogen peroxide stress. Reports suggest that *TSA1* also has a role in the oxidative DNA damage repair. Our results suggests that EPS treatment rescues yeast *tsa1∆* cells from peroxide induced stress. Mammalian peroxiredoxins have a positive effect on the cell growth, metabolism and immune functions. Their deficiency results in the elevated cellular oxidative stress that affects crucial signaling pathway and is implicated in neurodegenerative disorders, malignancies and inflammatory diseases^72^. Glutathione antioxidant mechanism is another first-line enzymatic antioxidant defense system present in the cells to overcome the oxidative stress. *GLR1* is the yeast homolog of mammalian glutathione reductase, localizes to both cytosol and mitochondria^73^. Glr reduces oxidized glutathione (GSSH) to reduced glutathione (GSH) and plays a role in maintaining GSH levels in the cells which in turn detoxifies superoxide and hydroxide radicals, thus protecting the cells from oxidative stress^74^. Our results show a high sensitivity of yeast *glr1∆* to H_2_O_2_ which was alleviated by pre-treatment with galactan EPS suggesting that galactan EPS might protect the cells that are deficient of *GLR1* from oxidative stress. Interestingly, glutathione reductase deficiency has been implicated in aging and age related metabolic, degenerative and cardiovascular disorders^75,76^. As shown in the Fig. 4A, *yhb1∆* was also protected by galactan EPS against H_2_O_2_ stress which suggests that galactan EPS protects the cells that are deficient of *YHB1* from oxidative stress. Yeast *YHB1* is a flavohemoglaobin that has been reported to protect the cells from nitric oxide stress^77^ and oxidative stress^78^. Evidence suggests that the human homolog of *YHB1* appears to rescue cells from alpha-synuclein toxicity which is a biomarker of Parkinson’s disease^79^. These previous findings and our results suggest that EPS may promote cell survival against the development of degenerative disorders.

To assess the level of ROS in the yeast mutants with the presence and absence of galactan under H_2_O_2_ stress, yeast cells were observed under fluorescence microscope and the intracellular oxidation level were calculated with spectrofluorometer^47,48^. Yeast mutants treated with H_2_O_2_ alone showed more number of green fluorescent cells compared to those pre-treated with galactan. (Fig. 4C). DCF fluorescence intensity increased by approximately 60%, whereas with galactan pre-treatment, it reduced to approximately 30% in H_2_O_2_ treated yeast mutants compared to WT and respective control (Fig. 4D). Both microscopic and spectrofluorometric results indicate an increased level of ROS in the cells that lack specific antioxidant genes compared to respective controls. Cultures pre-treated with galactan and then exposed to H_2_O_2_ showed a diminished fluorescence compared to those without galactan pre-treatment which suggests that galactan reduced the ROS induction H_2_O_2_ exposure in yeast mutant cells and promotes cell survival.

In all the above experiments, when the cells were washed before the addition of H_2_O_2_ showed no significant rescue (supplementary data) indicating that the ROS scavenging by galactan is due to direct scavenging in the medium rather than in the intracellular action.

### Galactan protects yeast cells from apoptotic cell death

**Figure 5:**
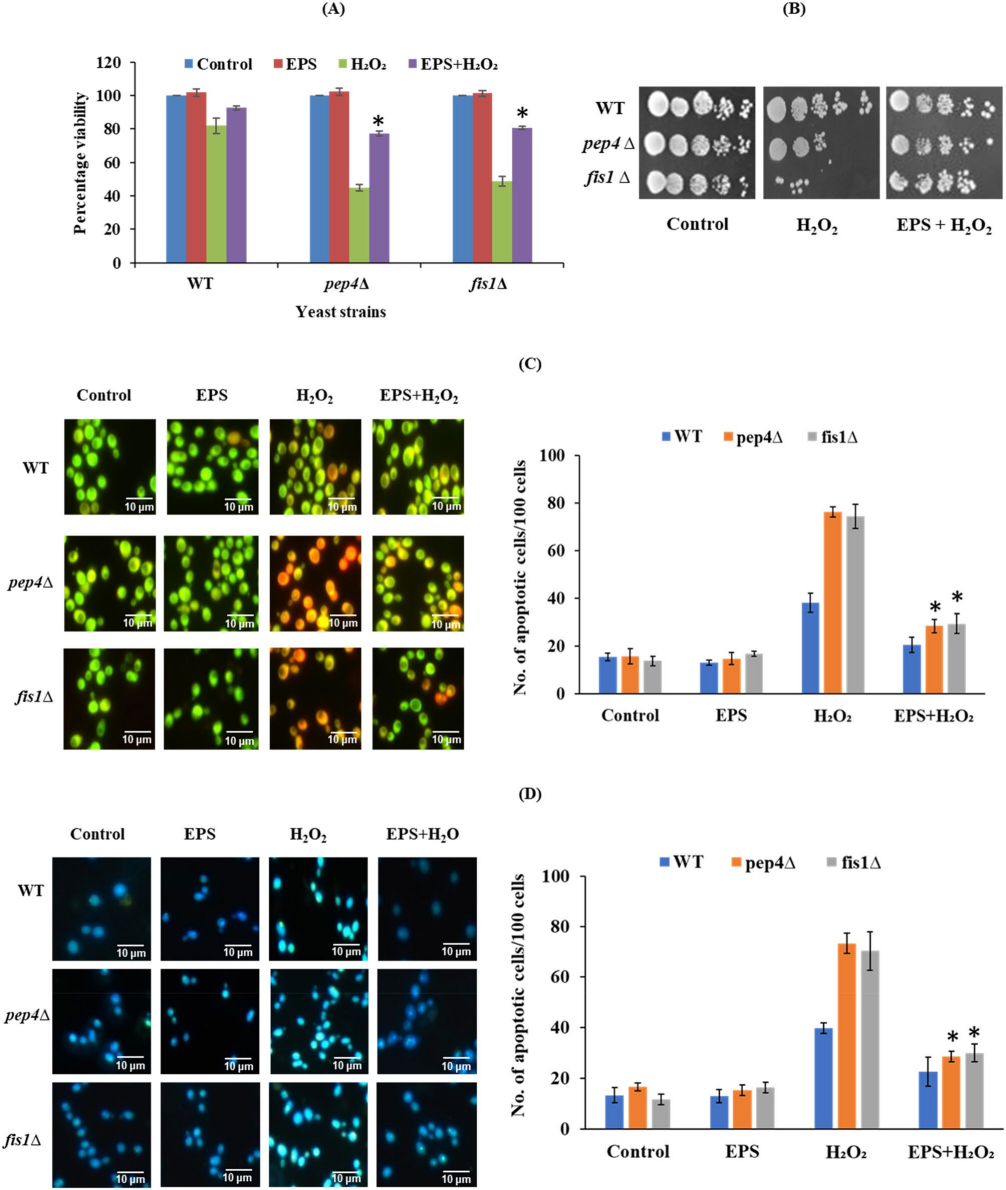
Anti-apoptotic activity of galactan EPS in yeast mutants (*pep4∆* and *fis1∆*). WT and mutants were pre-treated with or without EPS for 2 h followed by exposed to H_2_O_2_ for 1 h, later cells were serially diluted and performed (**A**) Viability and (**B**) Spot assay. (**C**) Detection of apoptotic markers: Yeast cells were treated with or without EPS for 2 h followed by treatment with or without H_2_O_2_ for 1 h. Cells were stained with fluorescence dyes specific to apoptosis and observed under a fluorescence microscope. And AO/EB staining: Chromatin condensation was observed by AO/EB staining and the number of apoptotic cells/100 cells were counted and plotted as a graph. (**D**) DAPI Staining: Nuclear fragmentation was observed by DAPI staining and the number of apoptotic cells/100 cells were counted and plotted as a graph. Data are mean ± SD of three independent experiments. ∗ represents *P* < 0.0001 a significant increase in EPS + H_2_O_2_ treated samples compare to those treated with EPS alone. Images shown are of at least three independent experiments.

To check if galactan has anti-apoptotic properties, we treated yeast anti-apoptotic gene deficient mutants (*fis1∆* and *pep4∆*) with galactan and exposed them to an apoptosis inducer, H_2_O_2_. While yeast Fis1 is a mitochondrial fission protein and prevents apoptosis, yeast Pep4 is a vacuolar aspartyl protease and protects the cells from acetic acid induced apoptosis^80,81^. CFU counts showed about 25–30% increase in the survival of both *pep4∆* and *fis1∆* cells when treated with galactan compared to the cells under apoptotic stress induced by 1 mM H_2_O_2_ (Fig. 5A). Spot assay results also showed a concordance with the CFU counts that showed better survival of galactan treated yeast anti-apoptotic mutants under apoptotic stress (Fig. 5B). Our results show that galactan protects yeast cells from apoptotic cell death induced by H_2_O_2_.

#### Galactan reduces chromatin condensation

In this study, we performed AO/EB staining of the galactan pre-treated yeast cells. The *pep4∆* and *fis1∆* cells with H_2_O_2_ alone mostly appeared yellow-orange compared to WT, and a notable reduction in the number of yellow-orange color cells was observed in the cells pre-treated with galactan and then, exposed to H_2_O_2_. This shows that the number of apoptotic cells significantly reduced in *pep4∆* and *fis1∆* cells upon galactan pre-treatment under H_2_O_2_ induced apoptotic stress (Fig. 5C). Our results suggest that galactan pre-treatment protected the yeast mutant cells from apoptotic cell death induced by H_2_O_2_,by reducing and chromatin condensation. AO/EB staining is a method used to detect apoptosis in both yeast and mammalian cells. Acridine orange is a cell-permeable dye that readily stains nucleic acids in both viable and non-viable cells, whereas ethidium bromide is a DNA intercalating dye that enters only when the cells are disintegrated or apoptotic or dead. This selectivity of AO/EB staining enables the detection of cells in three different phases i.e., viable cells with uniform green color in both nucleus and cytoplasm, early apoptotic cells with bright green colored nucleus due to chromatin condensation which stands out in the cytoplasm and late apoptotic cells which show yellow to orange/bright red color due to the entry of ethidium bromide into the cells^50,51^.

#### Galactan reduces nuclear fragmentation

Yeast WT and anti-apoptotic deficient mutants (*pep4∆* and *fis1∆*) pre-treated with or without galactan were exposed to H_2_O_2_ and subjected to DAPI staining. Yeast anti-apoptotic deficient mutant cells pre-treated with galactan showed lesser nuclear fragmentation and low intensity of DAPI, while those without galactan pre-treatment showed a significantly higher fluorescence and increased nuclear fragmentation (Fig. 5D)^52^. This indicates that that galactan can effectively alleviate the oxidative stress induced nuclear fragmentation leading to cellular disintegration.

Previously, it was shown in the transgenic mice that *Bifidobacterium breve* reduces apoptotic features like cell shedding in the intestinal epithelial cells which is mediated by the EPS present on the surface of the Bifidobacterium. The report shows a significant dose dependent reduction in cell shedding and the expression levels of apoptotic markers. Bifidobacterium EPS protected the cells from apoptic cell death by modulating both intrinsic and extrinsic apoptotic signaling pathways^82^. Our results, in agreement with the previous results, indicate that the galactan EPS protects the yeast anti-apoptotic gene deficient mutants *pep4∆* and *fis1∆* from the apoptotic stress induced by H_2_O_2_.

### Galactan extends CLS of *S. cerevisiae*

To assess the anti-aging activity of galactan we performed CLS assay with WT and yeast mutants (*sod2∆*, *tsa1∆* and *ctt1∆*)with galactan treatment. *SOD2* is a manganese SOD, localized to the mitochondrial matrix. The deletion of *SOD2* renders the cells highly sensitive to oxidative stress and a high mutation rate because the mtDNA is more accessible to ROS generated in the mitochondria^83^ and is one of the genes associated with the chronological life span of yeast. *TSA1* is a thioredoxin peroxidase (thiol-specific antioxidant) which is both cytoplasmic and ribosome associated. *TSA1* is involved in conferring the cells resistance against the oxidative stress induced by hydrogen peroxide and lack of *TSA1* is known to affect the lifespan of yeast cells. Another antioxidant mutant *ctt1∆* used in the CLS experiment lacks *CTT1*, the cytosolic catalase involved in the detoxification of hydrogen peroxide.

**Figure 6:**
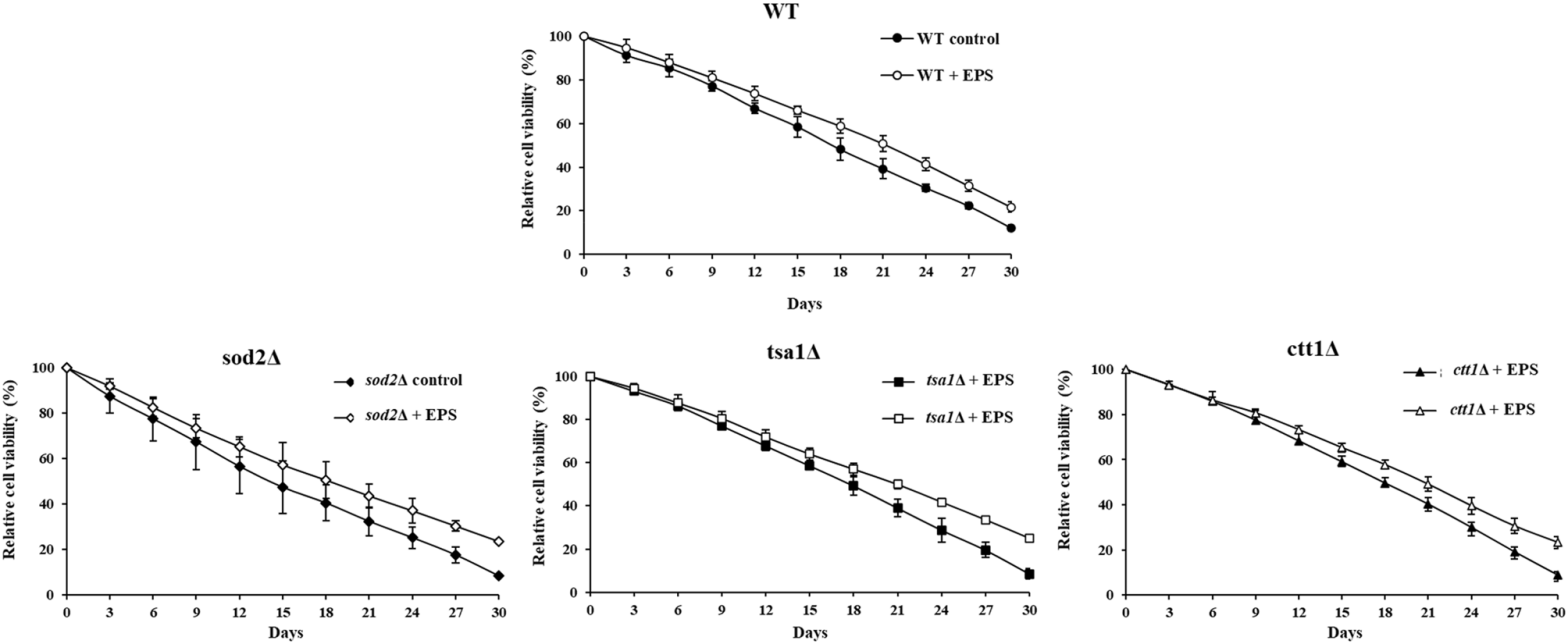
Anti-aging effect of galactan EPS by chronological lifespan assay (CLS) to yeast. WT and superoxide (*sod2∆*), peroxide (*tsa1∆*) and catalase (*ctt1∆*) mutants**.** Cells were grown in SD with or without EPS for 30 days, and CFU assay was carried out after every 3 days. Data are mean ± SD of three independent experiments.

Figure 6 shows the effect of galactan on CLS of the yeast mutant strains lacking anti-oxidant genes. While WT cells treated with galactan EPS showed ~ 10% increase in the viability, *sod2∆*, *tsa1∆* and *ctt1∆ ,* showed 15–20% increase in the viability (Fig. 6). Mitochondrial ROS leads to senescence and nuclear DNA damage, and *SOD2* is involved in scavenging ROS accumulation in mitochondria. *TSA1* and *CTT1* are involved in the detoxification of peroxide accumulated in the cytoplasm which is significantly higher in aging cells. Our CLS assay results indicate that EPS scavenges mitochondrial as well as cytoplasmic ROS buildup, prevents senescence or cell death-like features and extends the CLS in the yeast cells lacking *SOD2, TSA1* and *CTT1*^54^ .

Cellular oxidative stress is closely associated with the aging process and it greatly influences the onset of several age associated human diseases such as diabetes, cardiovascular diseases, neurodegenerative disorders and cancers. Oxidative stress and inflammation are two inter-linked events that play a major role in the pathology of many chronic diseases. Recent reports suggest a close link between the effect of gut microbiota on these age related disorders. Gut microbiome protects the intestinal epithelial lining from cellular damage induced by oxidative stress induced inflammatory pathways. Complex gut microbiota interacts with the ROS and the antioxidant defense system, helps in scavenging the free radicals and prevent inflammation. and may also regulate the oxidative state of the central nervous system through the production of neurotransmitters such as GABA, dopamine and serotonine. The microbiome also has immunomodulatory activity, prevents extensive colonization of the pathogenic microbes and provide immunity against many microbial infections^83^. Many microbial polysaccharides have been tested in different models and reported to have a high antioxidant potential, anti-inflammatory activity and cell protective functions evidenced by modulating the respective biomarker levels mediated by these exopolysaccharides^82,84^. These molecules have been found to have neuroprotective effect by preventing amyloid plaques and neuronal death in Alzheimer’s disease experimental models. Also, some of them have been shown to enhance SOD activity, reduce neurotoxic marker levels and inhibit dopaminergic neuron death in Parkinson’s disease experimental models.

Aging process is marked by elevated cellular oxidative leading to an increased accumulation of DNA damage, elevated mutation rate and decrease in cell viability. It is has been previously shown ROS induces apoptotic cell death in chronologically aging yeast cells and the supplementation of cells with the natural compounds can increase the longevity of the aging cells^47,85^. Our results on the antiaging effect exerted by galactan EPS from *W. confusa*, on the yeast *sod2∆*, *tsa1∆* and *ctt1∆* shows that galactan EPS protects the yeast cells from aging induced ROS and apoptosis, and increases their viability. The human homologs of these genes are implicated in providing protection against age related neurodegenerative disorders and cancers. Our results suggest that supplementation of galactan EPS may rescue the cells that lack these anti-oxidant defense genes from the age related cell death and reduce the risk of developing age related diseases.

## Conclusion

Oxidative stress is a cause of many unpropitious processes inside the cells which leads to various diseases and disorders. Galactan from *W. confusa* KR780676 *Weissella confusa* KR780676 exhibited pronounced antioxidant properties both in vitro and in vivo studies . This study clearly showed that galactan protected the yeast antioxidant mutants against the extracellular oxidative stress. Galactan also protected the anti-apoptotic deficient mutant cells from oxidative mediated apoptotic stress as evidenced by the decreased nuclear fragmentation. Galactan decreased ROS levels extending theirt life expectancy, shielding the cells from oxidative stress. Overall, the results prove that galactan has the ability to alleviate the oxidative stress in the medium by scavanging the free radicals. It would be interesting to test the antioxidant and anti-aging potential of galactan in higher eukaryotic cell models such as mammalian cell lines as well as animal models. Galactan is credited with strong prebiotic activity and antioxidant activity can play a significant role in reducing oxidative stress in the gut in addition to maintaining the gut homeostasis. With various technological properties credited to galactan such as strong emulsifying property, it would find immense application in the food and pharma industry as a natural functional ingredient.

## Supplementary Information

Supplementary Information.
