# Supplementary material for: Oxidative stress alleviating potential of galactan exopolysaccharide from Weissella confusa KR780676 in yeast model system
Source: Sci Rep. 2022 Jan 20;12:1089. doi: 10.1038/s41598-022-05190-2 (PMC8776969; doi:10.1038/s41598-022-05190-2)
Supplement: Supplementary file 1 — Supplementary Information. [file 41598_2022_5190_MOESM1_ESM.docx]

**Supplementary data:**

In *in vivo* antioxidant assay, yeast cells pre-treated with or without galactan EPS were washed and then, exposed to H_2_O_2_ followed by assessment of rescuing effect of galactan to yeast cells from oxidative stress in terms of viability.

**Observation:** Below figure shows that when the yeast cells pre-treated with galactan EPS were washed before the addition of H_2_O_2_ showed no significant protection from the oxidative stress. This reveals that large molecules of galactan EPS are not entering inside the yeast cells, rather it can protect the cells in the medium.

**
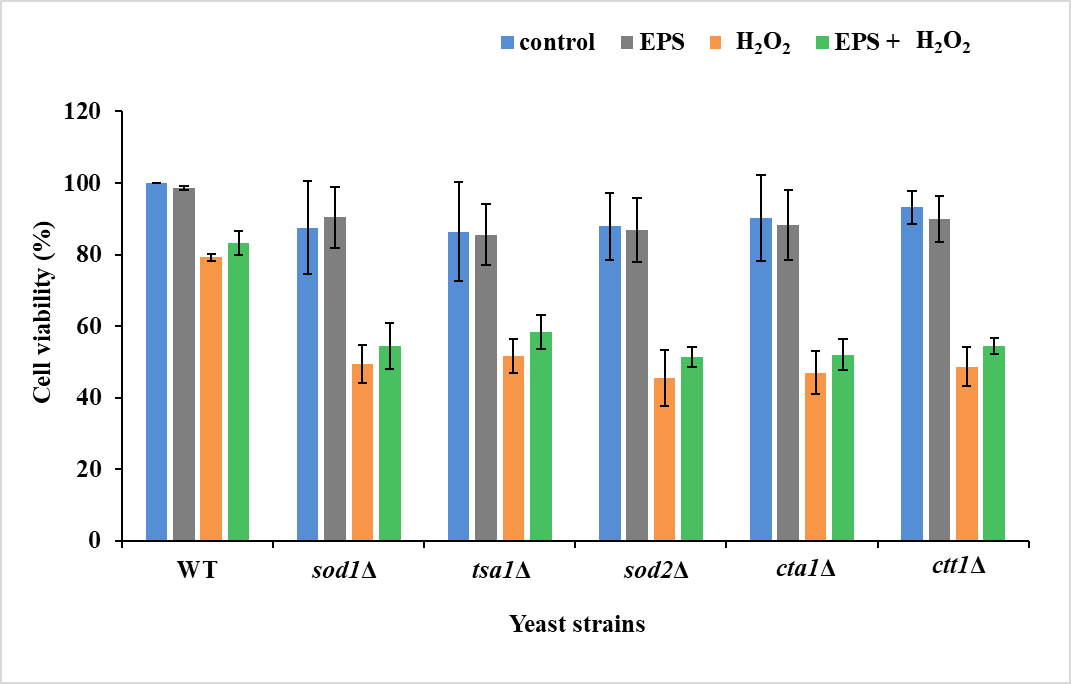
**

**Figure: Growth protection to *S. cerevisiae* gene deletion mutants by galactan EPS from H_2_O_2_ induced oxidative stress**. Viability assay: Cells pre-treated with and without galactan EPS for 2 h, then EPS is washed out from cells and cells incubated with or without H_2_O_2_ for 1 h. Then performed CFU assay.
